# Altered HIV-1 Viral Copy Number and Gene Expression Profiles of Peripheral (CEM CCR5+) and Mucosal (A3R5.7) T Cell Lines Co-Infected with HSV-2 In Vitro

**DOI:** 10.3390/v14081715

**Published:** 2022-08-04

**Authors:** Dipen Desai, Rajkumar Londhe, Madhuri Chandane, Smita Kulkarni

**Affiliations:** Division of Virology, ICMR-National AIDS Research Institute, Pune 411026, India

**Keywords:** HSV-2, HIV-1, co-infection, T-cell line, mRNA sequencing

## Abstract

Co-infecting pathogens have been speculated to influence Human Immunodeficiency Virus (HIV) disease progression. Herpes Simplex Virus Type-2 (HSV-2), another sexually transmitted pathogen, is commonly observed in individuals with HIV-1. Some clinical studies have observed an increase in HIV-1 viral copy number in HSV-2 co-infected individuals. In vitro studies have also demonstrated an increase in the expression of HIV-1 co-receptors on immune cells infected with HSV-2. Although both the viruses show distinctive persistent infection, the influence of HSV-2 on HIV-1 is poorly understood. Here we present a comparative analysis of primary CD4+ T-cells and four different T-cell lines (PM-1, CEM CCR5+, MOLT4 CCR5+, and A3R5.7) to assess the influence of HSV-2 co-infection on HIV-1 replication in vitro. Cell lines indicating significant changes in HIV-1 viral copy number [CEM CCR5+ (0.61 Log10), A3R5.7 (0.78 Log10)] were further evaluated for the infectivity of HIV-1 virions and the changes in gene expression profiles of HSV-2/HIV-1 co-infected and mono-infected cells, which were further confirmed by qPCR. Significant changes in NUP, MED, and VPS mRNA expression were observed in the gene expression profiles in co-infected CEM CCR5+ and A3R5.7 cells. In both cell lines, it was observed that the WNT signaling, PI3 kinase, apoptosis, and T-cell activation pathways were negatively affected in co-infected cells. The data suggest that HSV-2 infection of T-cells may influence the expression of genes that have been previously shown to affect HIV-1 replication in vitro. This idea needs to be explored further to identify anti-viral targets for HSV-2 and HIV-1.

## 1. Introduction

About 37.7 million people are currently suffering from AIDS, of which 1.7 million are children under the age of 15 years (UNAIDS, 2021). According to the NACO report 2020, the total number of people living with HIV/AIDS in India is 2.3 million, with 57,550 newly reported infections (NACO Technical Brief 2020 http://www.unaids.org/en/regionscountries/countries/India, accessed on 10 May 2022). Some clinical studies have speculated that HSV-2, another sexually transmitted pathogen, may be associated with HIV-1 infection. [1,2]. HSV-2, an alphaherpesvirus, belonging to the family Herpesviridae, causes skin abrasions and the formation of genital ulcers. Within the skin, HSV-2 encounters nerve endings and migrates to the regional neuronal ganglia, establishing a lifelong latent infection. The virus reactivates due to changes in immune responses and stress. Symptomatic infections of HSV-2 can be controlled by antivirals such as acyclovir, valacyclovir, and foscarnet [3,4].

Clinical studies undertaken to investigate the effect of HSV-2 and HIV-1 replication were inconclusive [1,5,6,7]. Some studies have indicated an increase in HIV-1 viral copy number in patients with HSV-2 co-infection, while others have reported no association between HSV-2 and HIV-1 infection. However, encouraging results have been reported in studies undertaken to assess the use of available antiviral therapy (acyclovir, valacyclovir) against HSV-2 in co-infected individuals. It has been also observed that the use of anti-HSV drugs was able to slow HIV-1 disease progression [5,8,9,10]. Similarly, in vitro co-culture studies conducted for understanding the interaction between the two viruses using dendritic cells and T-lymphocytes have shown that both HSV-2 and HIV-1 can be present inside a single cell [11]. Other studies have also reported that the HSV proteins play a supportive role in HIV-LTR-driven transcription. ICP-0, the transcriptional activator of HSV, cooperates with HIV-1 Tat to support HIV-1 transcription [11,12,13,14].

The HSV-2 genome encodes proteins required for its lifecycle [3,15] and evades the host immune system effectively by targeting host cellular processes [16,17]. On the contrary, HIV harbors the structural proteins and enzymes with specific functions such as reverse transcription, integration, viral protein cleavage, and maturation. The virus is therefore completely dependent on the host machinery for transcription, translation, assembly, and egress. Because of this dependency of HIV-1 on host proteins, the interaction between the virus and the host has been under constant investigation to understand factors that support or hinder virus replication [18]. Various in vitro studies have described Host Dependency Factors (HDFs) that are essential for HIV-1 to replicate efficiently within the infected host [19,20,21]. There are as many as 300 HDFs that have been identified by various studies affecting HIV-1 replication specifically within the immune cells [21]. Therefore, an influence on these HDFs by external factors such as a co-infecting pathogen such as HSV-2 may result in the exacerbation of HIV-1 replication [22]. Hence, it is important to investigate and assess the effect of HSV-2 proteins on HDFs within T-cells.

An in vitro study was therefore designed to investigate the effect of HSV-2 infection on HIV-1 replication and host gene expression. In this study, the ability of HSV-2 to infect four T-cell lines, primary CD4+ T-cells, and its influence on HIV-1 replication was assessed. The infectivity of the viral progeny generated from these cell lines was measured in TZM-bl cells. Furthermore, based on changes in HIV-1 viral copy number generated in these cell lines, two cell lines were selected for investigating the changes in the gene expression as a result of mono and/or dual infection. These results were further confirmed by real-time PCR.

## 2. Materials and Methods

### 2.1. Cell Lines

All T-cell lines (PM-1, MOLT4 CCR5+, CEMCCR5+, and A3R5.7) used in the study were procured from the National Institute of Health (NIH) under the AIDS Research Reference Reagent Program (ARRRP). Primary CD4+ T-cells were isolated using the negative separation method (RosetteSep, CD4+ T-cell separation kit) from the whole blood of two healthy donors. PM-1 and primary CD4+ T-cells express the CCR5 co-receptor naturally, while the CEM CCR5+ cells are transfected to constitutively express the CCR5 co-receptor. On the other hand, optimum levels of CCR5 co-receptor expression on MOLT4 CCR5+ and A3R5.7 cells are maintained by the use of geneticin. These cell lines were maintained in Roswell Park Medical Institute RPMI-1640 (Gibco, Waltham, MA, USA) medium supplemented with 10% heat-inactivated Fetal Bovine Serum (Hi-FBS, Gibco, Waltham, MA, USA) and 100 units/mL of penicillin and 100 μg/mL streptomycin (Gibco, Waltham, MA, USA) (R-10). The maintenance medium (R-10) for the MOLT4 CCR5+ and A3R5.7 cell lines required the addition of G418 (Sigma Aldrich, St. Louis, MO, USA) at a final concentration of 1 mg/mL for expression of the CCR5 co-receptor.

African Green Monkey Kidney Epithelial cell line (Vero) and the TZM-bl cell line were procured from the National Institute of Virology, Pune, and NIH-ARRRP, respectively. TZM-bl cell line is a genetically engineered JC53BL-13 clone. It expresses CD4, CCR5 and CXCR4 receptors along with a firefly luciferase reporter gene under the control of HIV-1 LTR. These cells were maintained in Dulbecco’s modified Eagle’s medium (DMEM) supplemented with 10% Hi-FBS (Gibco, Waltham, MA, USA), 100 units/mL of penicillin and 100 µg/mL streptomycin (Gibco, Waltham, MA, USA) [D-10]. The Vero cell line was used for the determination of HSV-2 load in stock cultures and co-infection experiments by plaque assay. Similarly, the TZM-bl cell line was used for assessing the infectivity of HIV-1 in the culture supernatants from the co-infection experiments.

### 2.2. Viruses

HSV-2 ATCC VR 734 strain was procured from American Type Culture Collection (ATCC). The virus was cultured and titrated in Vero cells as described earlier and the virus count was expressed as pfu/mL [23].

For preparing UV-inactivated HSV-2 virus, 6.0 mL of the viral stock was transferred to a 60 mm culture dish (NEST Biotechnology, Wuxi, China) and exposed to UV in the laminar airflow for 30 min by maintaining a distance of 12 cm between the UV lamp and the culture dish. As the volume of the virus suspension was reduced by half, it was made up to 6.0 m by adding a fresh culture medium (D-10). The culture dish was thoroughly washed to make sure all the area was rinsed while adding the fresh culture medium. The virus suspension was filtered using a 0.2 µ syringe filter (Acrodisc^®^Pall Scientific, Washington, NY, USA), aliquoted, and stored at −80 °C until further use. The infectivity of the virus was assessed by the plaque assay in the Vero cell line.

A CCR5 tropic, HIV-1_Ada5_, subtype B strain was procured from NIH under the ARRRP program [24]. The virus was cultured in activated Peripheral Blood Mononuclear Cells (PBMC) [25] and the stock was titrated in all T-cell lines used in the study.

### 2.3. Co-Infection of T Cell Lines with HSV-2 and HIV-1

Actively growing T-cell lines (PM-1, MOLT4 CCR5+, CEM CCR5+, A3R5.7) and primary CD4+ T-cells (4 × 10^6^ million cells) re-suspended in 200 μL of R-10 medium containing 8 μg/mL of polybrene (Sigma Aldrich, St. Louis, MO, USA) were exposed to either 0.5 MOI or 0.1 MOI of HSV-2. The virus was allowed to adsorb onto the cells for 2 h by incubating at 37 °C in a 5% CO_2_ incubator. Subsequently, the cells were washed thrice in R-2 medium (RPMI-1640 + 2% FBS), re-suspended in 200 μL of R-10 containing 8 μg/mL of polybrene, and exposed to HIV-1_Ada5_ (20 TCID_50_/mL) for 4 h in similar conditions. Post-infection, the cells were washed thrice with an R-2 medium. An aliquot from the last wash was collected to confirm the absence of the HIV-1 virus in the supernatant by p24 antigen ELISA. The cells were re-suspended in 4 mL of R-10 containing 8 μg/mL of polybrene and divided into two wells of a six-well plate (NEST Biotechnology, Wuxi, China). One well was terminated on the third day post-infection while the second well was terminated on the fifth day post-infection. The presence of infectious HSV-2 in the culture supernatants and infected cells was assessed by plaque assay and infectious center assay in Vero cells. The supernatants were also used for estimation of HIV-1 p24 antigen (ABL Inc., Rockville, MD, USA) and HIV-1 viral copy number (Abbott, Chicago, IL, USA). The infectivity of the HIV-1 virus was verified by infecting the TZM-bl cells and measuring the luminescence. The presence of viral proteins was also assessed using in-direct immunofluorescence (IFA). Appropriate controls (cells infected with HSV-2 alone, HIV-1 alone, and mock-infected cells) were included in each experiment.

### 2.4. HSV-2 Plaque Assay

HSV-2 count in the supernatants was measured by plaque assay as described earlier {23]. Briefly, supernatants from the co-infection assay were added to a pre-seeded monolayer of Vero cells (0.3 × 10^6^ cells/well) in a 24-well plate. The virus was allowed to adsorb for 2 h at 37 °C in a 5% CO_2_ incubator, following which a 1:1 mixture of D-10 and 2% carboxymethyl cellulose (CMC, Sigma Aldrich, St. Louis, MO, USA) was overlayered onto the monolayer and the plates were incubated for 5 days at 37 °C in 5% CO_2_ incubator. The monolayer was washed with sterile PBS and the presence of plaques was assessed by staining the monolayer using amido black solution stain (Sigma Aldrich, St. Louis, MO, USA). The count obtained was expressed as pfu/mL.

### 2.5. HSV-2 Infectious Center Assay for Intracellular HSV-2

The aliquots of infected T-cells stored in the liquid nitrogen were revived following the standard protocol and the assay was performed as described earlier [26] with minor modifications. Briefly, the cells were washed thoroughly to remove any cell-free HSV-2 that may be present and the number of live cells was estimated using the Neubauer’s chamber. The cells were re-suspended at a density of 2500 cells/mL in a 1:1 mixture of D-10 and R-10 medium. The cells were incubated at 37 °C in a 5% CO_2_ incubator for 2 h. The T-cell suspension (100 μL) was then added to 900 μL of a 1:1 mixture of D-10 and sterile 2% carboxy methyl cellulose (CMC). This 1 mL was overlaid onto a monolayer of Vero cells pre-seeded in a 24 well plate (Corning, NY, USA). The plate was then centrifuged at 500 rpm for 5 min to allow the infected T-cells cells to settle over the Vero cells. The plate was removed from the centrifuge carefully and incubated in a 5% CO_2_ incubator at 37 °C for 5 days. On the 5th day, the formation of plaques on the Vero monolayer was assessed as mentioned before. The number of infectious centers formed was calculated and represented as infectious centers/2 × 10^6^ cells/mL.

### 2.6. Luminescence-Based Assay for Cell-Free HIV-1 Quantification

TZM-bl cells were pre-seeded in a 96-well flat-bottom plate (Corning, NY, USA) at a density of 10,000 cells/well in a D-10 medium. The next day, the medium was changed and 100 μL of fresh D-10 medium containing 25 μg/mL of DEAE-Dextran (Sigma Aldrich, St. Louis, MO, USA) was added along with 100 μL of culture supernatants from the co-infection assay. The plate was incubated at 37 °C in a 5% CO_2_ incubator for 48 h. Post incubation, 120 μL of the medium was removed and 50 μL of Britelite^TM^ plus Reporter Gene Assay reagent (PerkinElmer, Waltham, MA, USA) was added and the plate was incubated for two mins at RT. The substrate and the medium were thoroughly mixed and 100 μL of the mixture was transferred to an Optiplate (Corning, NY, USA). The luminescence was measured in Victor3^TM^ luminometer (PerkinElmer, Waltham, MA, USA) and expressed as Relative Luminescence Units (RLUs). Uninfected TZM-bl cells were also maintained as a control.

### 2.7. In-Direct Immunofluorescence Assay

For indirect immunofluorescence assay, the cell pellets were loaded onto poly l-lysine coated coverslips. After drying, the cells were washed and fixed with 4% paraformaldehyde (Sigma Aldrich, St. Louis, MO, USA) for 10 min. The fixed cells were washed with PBS and permeabilized with PBS containing 2.0% BSA and 0.1% Triton X-100 for 30 min. The cells were then treated with HSV-2 gB (glycoprotein B) mouse monoclonal antibody (Sigma Aldrich, St. Louis, MO, USA) and/or with HIV-1 positive human serum in 2.0% BSA and then incubated overnight at 4 °C. The cells were washed thrice with PBS and subsequently treated with the secondary antibody at RT for 1 h. Anti-human FITC conjugated secondary antibody (Sigma Aldrich, St. Louis, MO, USA) was used for HIV-1 while a Vectafluor signal enhancement kit was used for HSV-2 staining. The cells were mounted on grease-free glass slides using Prolong^®^ GOLD anti-fade reagent (Invitrogen, Carlsbad, CA, USA) containing DAPI and visualized using the Olympus X171 microscope with a Cool LED filter. The images were captured and merged using the Cell F software.

### 2.8. Gene Expression Analysis

RNA sequencing was carried out to investigate the changes in the gene expression profiles of T-cell lines (CEM CCR5+ and A3R5.7) infected with HIV-1 alone or co-infected with HSV-2. The assay was carried out as described in co-infection experiments i.e., T-cell lines were infected with HSV-2 at 0.5 MOI for 2 h and then with HIV-1 using 20 TCID50/mL dose for 4 h. The cells were washed twice with PBS kept at 4 °C and 1 mL of RNA stabilizing reagent (Invitrogen, Carlsbad, CA, USA) was added to each tube. The cells were stored at 4 °C overnight followed by storage at −20 °C indefinitely until the RNA extraction and sequencing. Before RNA sequencing, the cells were thawed and the RNA was extracted using the RNeasy^TM^ mini kit, (Qiagen, Hilden, Germany) and stored at −80 °C until further use. The quantity and quality of RNA were analyzed by Qubit^®^ Fluorometer using the Quant-iTTM RiboGreen^®^ RNA assay kit and Agilent 2100 Bioanalyzer. After quality control analysis and library preparation, the library was sequenced on Illumina NextSeq500 using 2 × 75 bp paired-end sequencing with 40 million reads per sample. FastQC was carried out to assess the quality of the obtained reads. The sequences were processed to remove the adaptors and trimmed to obtain good-quality bases. The reads were aligned to the reference human genome hg- 19 (GCA_000001405.1) using HT-seq and mapping of the reads was carried out using Bowtie. The read counts for each of the genes were read using Cufflinks and CuffDiff, and the differential gene expression was assessed by Dseq and EdgeR software using the R-studio interface. The required programs were downloaded from the website (http://bioconductor.org/packages (accessed on 30 November 2017)). The reads per fragment per million (RPKM) counts were used to calculate log_2_ fold change (Log_2_FC). The heatmaps and the volcano plot were generated using ggplots2 in R-studio.

### 2.9. Real-Time PCR Assay

Based on the gene enrichment analysis and gene ontology, representative genes were selected for qPCR analysis using the SyBr Green method. The primers used (Table S1) were synthesized at Eurofins, Germany. The real-time PCR was carried out on the 7500 HT Real-time PCR thermal-cycler (Applied Biosystems, Waltham, MA, USA) using the GoTaq qPCR master mix (Promega, Madison, WI, USA) following the manufacturer’s instructions. ß-actin gene was used as endogenous control and the analysis was carried out using the ΔΔCt method to assess the changes in the gene expression.

### 2.10. Statistical Analysis

The data were analyzed by two-way ANOVA using Graphpad Prism 5^TM^. All data represent the mean ± SD of three independent experiments unless stated otherwise. Gene expression data and gene ontology data calculated *p*-values and False Discovery Rate (FDR) using Fischer’s exact test as per the software’s algorithm.

## 3. Results

### 3.1. T-Cell Lines Exhibited Differences in the Extracellular HIV-1 but Not HSV-2 Viral Copies Released in Culture Supernatants

This study aimed at identifying a suitable T-cell line model for studying HSV-2 and HIV-1 interactions in vitro. We assessed four T-cell lines (CEM CCR5+. A3R5.7, MOLT4 CCR5+, and PM-1) for their ability to sustain HSV-2 and HIV-1 infection. In a previous study on two of the cell lines (CEM CCR5+ and MOLT4 CCR5+), we observed that the cells differently expressed mRNA for Herpes Virus Entry Mediators (HVEM) receptors required for HSV-2 entry in T-cell lines [22]. Therefore, we initiated the experiments with four cell lines. We used HIV-1 subtype B virus as many previous studies had focused on the HIV-1 subtype B strain [11,12,13,22] mainly due to its prevalence in the US and European countries, where exhaustive research in this field has been underway. HSV-2 ATCC VR-734 strain is a typed virulent strain which we have used in our previous study on a T-cell line [23] and hence these two strains were used.

**Figure 1:**
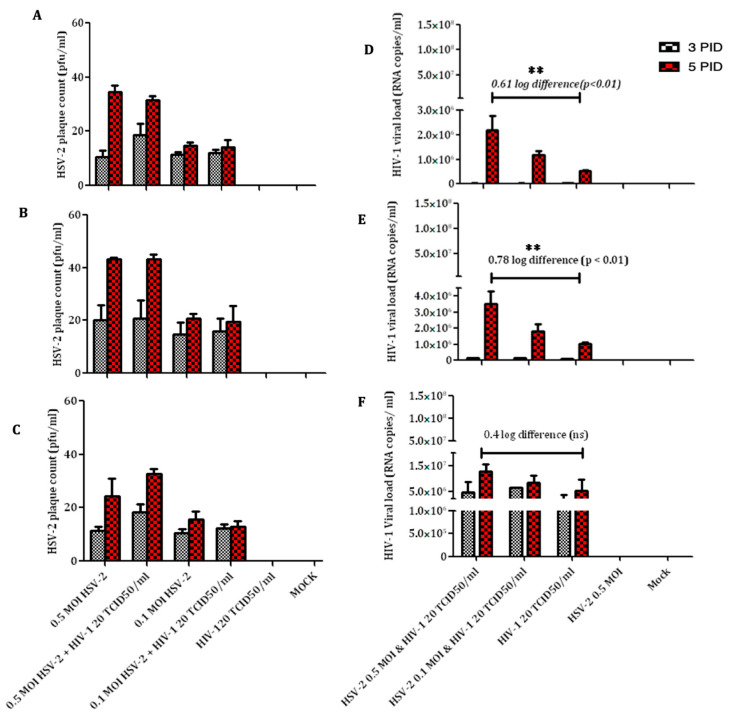
HSV-2 and HIV-1 viral copies released in the culture supernatants of T-cell lines. HSV-2 virus counts expressed as pfu/mL in CEM CCR5+ (**A**), A3R5.7 (**B**) and primary CD+ T-cells (**C**) in co-infection assays. Plaques were counted using Vero cell line. HIV-1 viral copy numbers in culture supernatants were assessed by automated Abbott M2000sp and M2000rt in CEM CCR5+ (**D**), A3R5.7 (**E**), and primary CD4+ T-cells (**F**). Data represent mean ± SD of three independent assays. ** indicates significant difference with *p* value < 0.01.

Plaque assay was performed to assess the number of extracellular HSV-2 viral particles present in the culture supernatant generated from co-infection experiments. A dose-dependent increase in HSV-2 was observed in all the cell lines studied (Figure 1A–C and Supplementary Figure S1A,B). Although insignificant, a two-fold increase in HSV-2 viral particles was observed in the supernatants collected on the fifth day compared to those collected on the third day post-infection. Furthermore, no significant differences were seen between the HSV-2 virus particles generated from HSV-2 and HIV-1 co-infected cells and cells infected with HSV-2 alone. HSV-2 virus was not detected in HIV-1-infected and mock-infected cells.

The extracellular HIV-1 viral copy number released in the culture supernatants in the co-infection experiments was assessed using the automated system (Abbott M2000sp and M2000rt) and the data were expressed as RNA copies/mL (Figure 1D–F). Significant differences in HIV-1 viral copy number (0.61 Log_10_ copies/mL and 0.78 Log_10_ copies/mL, *p*-value < 0.01) were observed in HSV-2 co-infected cells in CEM CCR5+ (Figure 1D) and A3R5.7 (Figure 1E) cell lines, respectively. A dose-dependent increase in HIV-1 viral copy number was also observed in these two cell lines when co-infected with HSV-2 at different MOIs. However, the HIV-1 viral copy number in A3R.7 cells co-infected with HSV-2 and HIV-1 was slightly higher (4 × 10^6^ RNA copies/mL) as compared to co-infected CEM CCR5+ cells (3 × 10^6^ RNA copies/mL). In primary CD4 + T-cells, higher HIV-1 viral copy numbers were observed in culture supernatants co-infected with HSV-2 (15 × 10^6^ RNA copies/mL) as well as in cells infected with HIV-1 alone (8.3 × 10^6^ RNA copies/mL) (Figure 1F). However, this difference was not significant. The difference in HIV-1 RNA copies/mL in the T-cell lines was in the order A3R5.7 < CEM CCR5 < PM-1 < MOLT4 CCR5+ cells, respectively. Taken together, the data suggest that CEM CCR5+ and A3R5.7 T-cell lines can be further used as models to study HIV-1 and HSV-2 co-infection in vitro.

### 3.2. UV-Inactivated HSV-2 Negatively Impacts HIV-1 Replication in the Two Candidate T-Cell Lines

**Figure 2:**
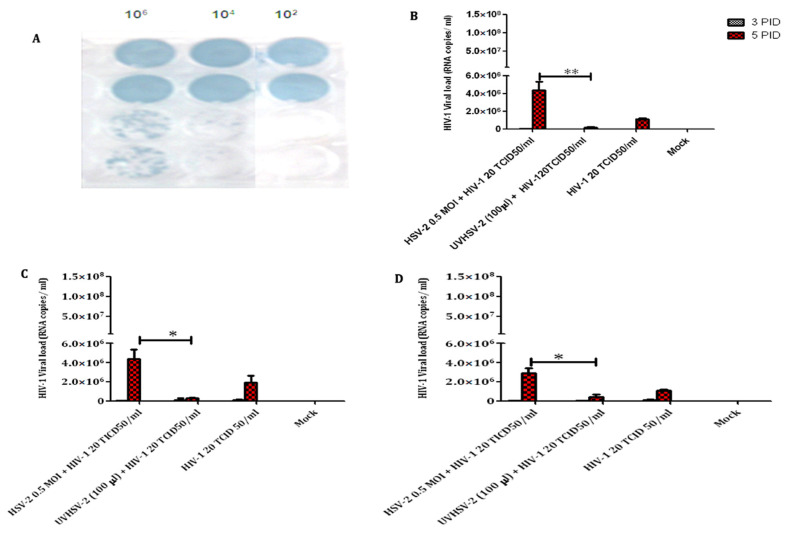
Effect of UV-inactivated HSV-2 on HIV-1 viral copy number in T-cells in vitro. Vero monolayer infected with UV-inactivated HSV-2 (top) and live replication-competent HSV-2 (bottom) (**A**). HIV-1 viral copy number/mL in culture supernatants of T-cell lines; CEM CCR5+ (**B**), A3R5.7 (**C**) and primary CD4+ T-cells (**D**) in co-infection assays. HIV-1 viral copy numbers were estimated using the automated Abbott Real-Time Platform. Data represent mean ± SD of two independent assays. * indicates significant difference with *p* value < 0.05 while ** indicates *p* value < 0.01.

To assess whether live HSV-2 is required to affect HIV-1 replication in T-cell lines, UV-inactivated HSV-2 was generated for co-infection experiments. It was confirmed that the UV-inactivation suppressed the infectivity of the HSV-2 virus as no plaques were generated on a monolayer of Vero cell line even at the highest concentration of the virus (Figure 2A). Subsequently, the generated UV-inactivated virus was used in co-infection assay in CEM CCR5+ and A3R5.7 cell lines as well as primary CD4+ T-cells. It was observed that the extracellular HIV-1 viral copy number in the culture supernatants of co-infected cells significantly decreased as compared to cells infected with HIV-1 alone and those co-infected with replication-competent HSV-2 (Figure 2B–D). These data suggest that UV inactivated HSV-2 may have a negative impact on HIV-1 replication and the changes in gene expression due to UV-inactivated HSV-2 need to be further explored.

### 3.3. Candidate T-Cell Lines May Harbor HSV-2 Intracellularly

**Figure 3:**
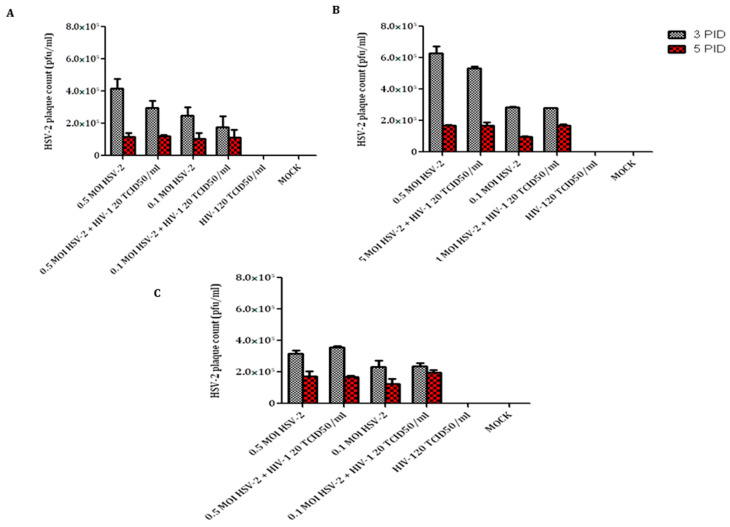
Infectious centers obtained by co-culturing infected T-cells with Vero cells: Plaque counts were obtained by exposing Vero monolayers to T-cells CEM CCR5+ (**A**), A3R5.7 (**B**), and primary CD4+ T-cells (**C**) infected with HSV-2 and/or HIV-1. Results for A3R5.7 and CEM CCR5+ represent data from three independent assays for T-cell lines and two independent donors for primary CD4+ T-cells.

Infectious center assay was performed to assess whether CEM CCR5+ and A3R5.7 T-cell lines harbor infectious HSV-2 intracellularly (Figure 3). In this assay, infected T-cells were washed and added to a monolayer of Vero cells to assess the generation of plaques. In both the T-cell lines, a higher number of infectious centers were observed in cells collected on the third day post-infection compared to the fifth day and were also dose-dependent. The A3R5.7 cells showed presence of higher number of infectious centers (6.2 × 10^5^ infectious centers/2 million cells) compared to CEM CCR5+ (4.2 × 10^5^/2 million cells) and primary CD4+ T-cells (3.12 × 10^5^/2 million cells). The presence of HSV-2 and HIV-1 in the infected cell lines was also visualized by indirect immunofluorescence assay, represented in Figure S2.

### 3.4. Infectivity Assay for HIV-1

**Figure 4:**
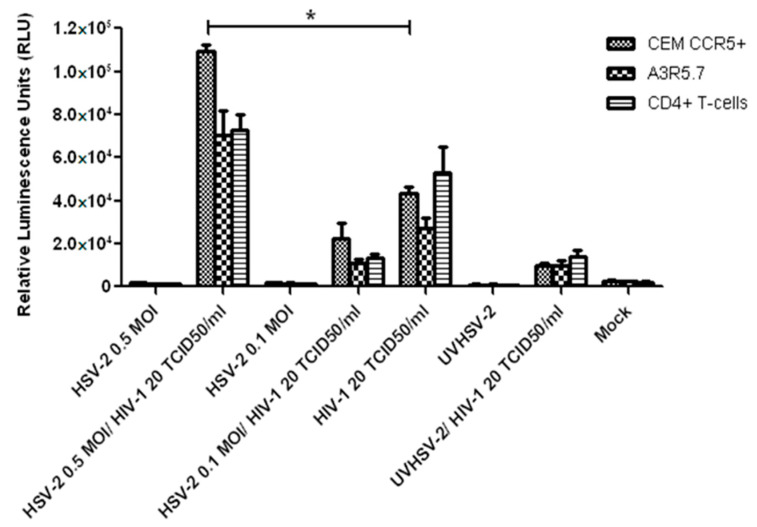
Infectivity of HIV-1 produced in culture supernatants of co-infected T-cells: HIV-1 virus in culture supernatants collected from co-infection assays were used to infect TZM-bl cells. The luminescence was measured using Victor 3 (Perkin Elmer, Waltham, MA, USA) luminometer and the results were expressed as Relative Luminescence Units (RLU). Data represent mean ± SD of three independent assays. * indicates *p*-value < 0.05.

To test the infectivity of the HIV-1 virus produced in co-infection assays, culture supernatants were added onto TZM- bl cells, and the growth of HIV-1 was expressed as Relative Luminescence Units (RLUs) (Figure 4). In all the supernatants tested, the RLUs produced by mock, HSV-2-infected (both 0.1 and 0.5 MOI), and UVHSV-2 were comparable to the un-infected TZM-bl cells. CEM CCR5+ and A3R5.7 cells showed a significant increase of 2.51-fold (RLU = 109,107.33) (*p*-value < *0.05*) and a 2.6-fold (RLUs: 70,007.67) (*p*-value < *0.05*) in HIV-1 infectivity, respectively, when compared to their respective HIV-1 controls (RLU = 43,449.78 and RLUs: 26,894.89, respectively). However, in primary CD4+ T-cells, the increase in the HIV-1 infectivity in co-infected cells was 1.37-fold (RLUs = 72,672) compared to in HIV-1 infected cells (RLUs = 52,841.89) and was not significant. These results suggest that HIV-1 infectivity may be higher in virus generated from immortalized cell lines compared to primary CD4 + T-cells in vitro.

### 3.5. Gene Expression Profile

RNA sequencing was carried out using RNA extracted from mono (HSV-2 or HIV-1) and dual (HSV-2/UV-HSV-2 and HIV-1) infected A3R5.7 and CEM CCR5+ cells. RNA sequencing was carried out from over 10 different infection conditions (five for each cell line). These consisted of HSV-2 and HIV-1 co-infected cells (A_HI and C_HI), HSV-2 infected cells (A_H and C_H), HIV-1 infected cells (A_I and C_I), UV-HSV-2 and HIV-1 co-infected cells (A_UI and C_UI) and mock infected cells (A_CC and C_CC). Post sequencing, the raw sequenced reads were initially checked for quality using FastQC/MultiQC. It was observed that the majority of the sequences had a length of 75–80 bp. The relative GC-content varied between 40–60% with 90% of the sequences having a PHRED (Phil’s Read Editor) quality score of 30 (data not shown). The number of paired sequenced reads generated for each condition analyzed is summarized in Table 1.

The raw read count obtained from HTSeq was used to study variations in the gene expression in various groups using DESeq2. For analysis, only genes expressed with a *p*-value of <0.05 and false discovery rate (FDR) adjusted *p* values ≤ 0.05 were selected and the total number of genes fitting this inclusion criterion are summarized in Table 2. Furthermore, the table also shows the total number of genes expressed only in either of the conditions followed by number of genes upregulated or downregulated with >1.5 log_2_ fold change. The read count obtained per gene was converted to transcripts per million (TPM) for calculating the fold change. The complete dataset of differential gene expression of the four groups assessed is compiled as spreadsheets and included in the Supplementary Material (Sheet S1–S4).

**Figure 5:**
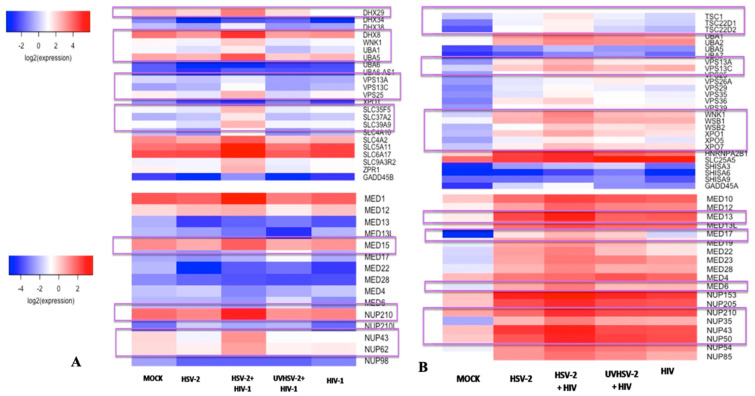
Heatmap of differentially expressed genes in infected T-cell lines. Log_2_ (RPKM) values of T-cell lines (A3R5.7 and CEM CCR5+) infected under various conditions were used to draw a heatmap using the ggplots2 program on R-studio. (**A**) Heatmap generated for A3R5.7 cells (**B**). Heatmap generated for CEM CCR5+ T-cell line.

The log_2_ reads per kilobase of transcript, per million mapped reads (RPKM) values calculated from the read counts obtained for various infection combinations were compiled together and used to create a heatmap to assess the changes in gene expression of a group of genes in the two T-cell lines. It was observed that the expression of NUP (nucleoporins) and MED (mediator complex) genes were upregulated in co-infected cells in comparison to mono-infected A3R5.7 or CEM CCR5+ cells (Figure 5). Amongst the NUP gene, NUP210 and NUP50 were upregulated in both the co-infected cell lines. Additionally, NUP 43 and NUP 62 mRNA were also upregulated in co-infected CEM CCR5+ cells. An increase in the expression of Tuberous sclerosis protein complex (TSC1 and TSC2) mRNA was observed in CEM CCR5+ cells co-infected with HSV-2 and HIV-1. Expression of the Vacuolar Protein Sorting (VPS) mRNA was also increased in both the cell lines co-infected with HSV-2 and HIV-1. Similarly, an increase in the expression of the exportin (XPO) mRNA was also noted in these cells. The data are represented as a heat map as shown in Figure 5 However, mRNA expression of the Ubiquinol Cytochrome C-Oxidase complex (UQCR) was fairly constant in both the cell lines (data not shown).

When analyzing the mRNA data on CD cell surface markers of the two cell lines, upregulation of CD1c, CD164, CD44, CD46, CD47, CD86, and CD96 genes occurred in A3R5.7 cells co-infected with HSV-2 and HIV-1 when compared to those infected with HIV-1 alone. On the other hand, in CEMCCR5+ co-infected cells, CD163L1, CD22, CD38, CD46, and CD86 were upregulated while CD80 and CD164 were downregulated when compared with cells infected with HIV-1 alone (Figure S3).

**Figure 6:**
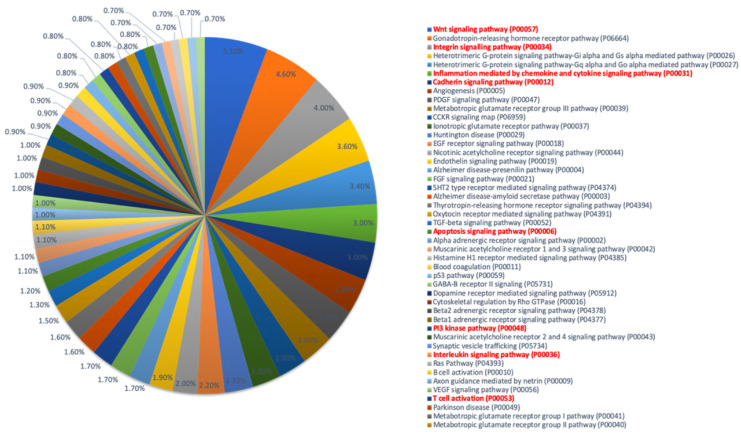
Gene Ontology pathway enrichment analysis in co-infected A3R5.7 cells. List of pathways downregulated in A3R5.7 cells infected with HSV-2 and HIV-1 based on gene enrichment analysis with a *p* < 0.05 and FDR < 0.05.

**Figure 7:**
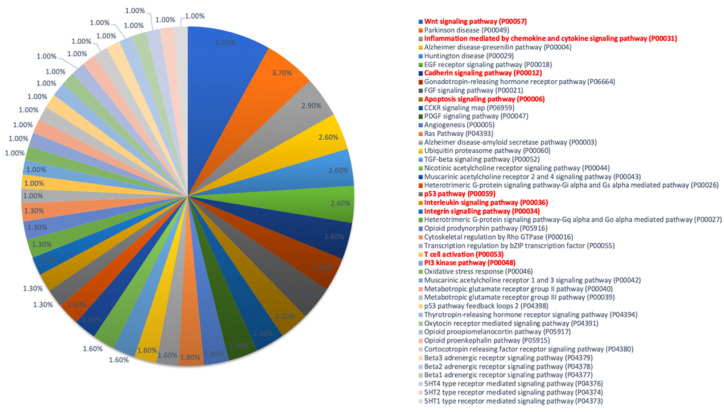
Gene Ontology pathway enrichment analysis in co-infected CEM CCR5+ cells. List of pathways downregulated in CEM CCR5+ cells infected with HSV-2 and HIV-1 based on gene enrichment analysis with a *p* < 0.05 and FDR < 0.05.

Gene enrichment and ontology were carried out using the Gene Ontology resource database (www.geneontology.org, accessed on 10 May 2022) to assess the pathways involved. The pathways enriched for A3R5.7 and CEM CCR5+ are shown in Figure 6 and Figure 7 respectively. The WNT signaling pathway was observed to be commonly affected in both the T-cell lines. Other pathways negatively affected d in both the cell lines included the inflammation mediated by cytokine and chemokine signaling, apoptosis signaling pathway, p53 signaling, PI3K signaling, and interleukin signaling pathway. These results indicate that HSV-2 infection of T-cell lines in vitro may aid HIV-1 replication by affecting immune signaling mechanisms.

### 3.6. Real-Time PCR

To confirm the results of the Next Generation Sequencing (NGS), a total of 15 genes (14 test and one control (GAPDH) genes) were assessed by quantitative PCR. RNA extracted from CEMCCR5+ and A3R5.7 cells used in co-infection assays was reverse transcribed using the first-strand cDNA synthesis kit and the relative gene expression of the selected genes was assessed in groups using the ΔΔCT method. The data obtained are presented in Table 3. A positive number indicates increased mRNA expression of the gene in the first condition. In A3R5.7 cells, it was observed that all the genes tested were upregulated in co-infected cells except XPO5 and TSG101. Similar observations were also noticed in the NGS data. The mRNA expression of most of the genes assessed was considerably reduced in UVHSV-2 and HIV-1 co-infected cells, except NUP210, suggesting that UV-inactivated HSV-2 may be affecting only certain cellular processes. In CEM CCR5+ cells, VPS mRNA (VPS13C and VPS25) and TSC 1 were upregulated in co-infected cells. Considerable changes were also noticed in the expression of XPO and NUP proteins in these cells. In UVHSV-2 and HIV-1 co-infected CEM CCR5+ cells, XPO5, NUP210, VPS13C, VPS 25, and TSC1 were upregulated compared to cells infected with HIV-1 alone. These results indicate that co-infection of HSV-2 may affect different pathways in mucosal and peripheral T-cell lines in vitro.

## 4. Discussion

The aim of this study was to investigate the effect of HSV-2 infection on gene expression of T-cell lines and its repercussions on HIV-1 replication. In co-infection assays, we observed differences in HIV-1 replication only in two cell lines, CEMCCR5+ and A3R5.7. These differences may be due to how the CCR5 co-receptor is expressed on these T-cells. Although A3R5.7 cells require similar conditions for infection to MOLT4 CCR5+, it was observed that A3R5.7 cell lines displayed higher HIV-1 replication than MOLT4 CCR5+ cells. A3R5.7 cells also express the α4β7 (mucosal integrin) receptor known to facilitate entry of HIV-1 into the mucosal T-cells, and that HIV-1 gp120 has more affinity toward the α4β7 receptor compared to the CD4 receptor [27]. Furthermore, we showed that a replication-competent HSV-2 is required for affecting HIV-1 replication, as a decrease in the HIV-1 viral copy number was observed in UV-HSV-2 and HIV-1 co-infected cells when compared to cells co-infected with wild-type replication competent HSV-2 and HIV-1. Although the mechanism responsible for this unexpected result was not further investigated in this study, previous studies have observed that tegument proteins of HSV-2 that sabotage the host replication machinery may also block many of the host response pathways [28,29,30], which could be a plausible reason for the reduction in the HIV-1 viral copy number due to UV-inactivated HSV-2 [31]. One of the tegument proteins is the virus–host shutoff RNases (VhsRNAses). This is a non-specific tegument protein that HSV-2 uses to regulate the expression of early and late genes in the HSV-2 lifecycle. HSV-2 achieves this by non-specifically blocking all RNA translation by degrading the host cellular mRNA. This protein is a characteristic of various α and γ− herpes viruses [32].

It has been reported previously that HSV-2 released in culture supernatant is significantly lower from T-cells compared to epithelial cells [23]. Using experiments similar to those used by Dutta and Myrup [26], it was observed that numbers of intracellular HSV-2 virus particles were significantly higher in T-cells on the third day post-infection as compared to the fifth day. Our previous study on the growth kinetics of HSV-2 has shown that T-cells harbor higher HSV-2 virus particles until the fourth PID [23]. A study by Vanden and colleagues reported that intracellular HSV-2 induces caspase-9-mediated apoptosis in T-cells, triggering cell death [33]. Similarly, on assessing the infectivity of the HIV-1 virus released in the culture supernatants using the TZM-bl cell line, we found that the HIV-1 progeny from co-infected CEM CCR5+ and A3R5.7 cells replicated productively, yielding a 2.5- and 2.6-fold increase in infectivity, respectively, as compared to cells infected with HIV-1 alone. However, the yield of HIV-1 progeny from co-infected primary CD4+ T-cells was less (1.37-fold increase). These data suggest that HIV-1 viral progeny in co-infected CEM CCR5+ and A3R5.7 cells was more infective than primary CD4+ T-cells.

In both the cell lines, the WNT signaling pathway was found to be negatively affected. Other pathways identified in both the cell lines included the inflammation mediated by cytokine and chemokine signaling, apoptosis signaling pathway, p53 signaling, PI3K signaling, and interleukin signaling pathway. Many of these pathways are interconnected with various branch points related to apoptosis, AKT activation, control of autophagy, and aiding cell survival and metabolism [34]. Although previous studies have shown that WNT signaling inhibits HIV replication in macrophages and T-cells [35], it has also been speculated that HIV-1 has evolved mechanisms (Vpu and Tat protein) to combat this effect [36]. Furthermore, the WNT pathway also influences the p53 pathway for further activation/suppression of the mTORc1 pathway mediated via the TSC-2 (Tuberous Sclerosis protein-2) and controls AKT activation, thereby dictating cell survival [34]. These data therefore suggest that an increase in HIV-1 viral copy number in the in vitro co-infection experiments observed in this study may be a result of the extended survival of the T-cells due to HSV-2.

The RNA sequencing analysis carried out in this study observed upregulation of many genes previously identified as important for HIV-1 replication in immune cells, especially T-lymphocytes. The studies carried out by Brass et al. and Zhou et al. demonstrated that the mediator complex is essential in HIV-1 replication [19,21]. In this study, we observed overexpression of mRNAs of mediator complex proteins in A3R5.7 cells co-infected with HSV-2 and HIV-1. Amongst these, MED12 and MED13 are involved in the WNT signaling pathway regulated through the β^−^ catenin pathway. Earlier studies have speculated that MED12 might repress the WNT signaling pathways [37]. However, the impact exerted by MED12 on WNT signaling depends upon other mediators interacting with MED12 and can form either a repressing or an activating complex [38]. On the other hand, MED1 and MED 15 were upregulated in the HSV-2 and HIV-1 co-infected cells CEM CCR5+ T-cells. Studies by Konig et al. (2008) have identified these proteins as responsible for HIV-1 replication [20]. MED15 also plays an important role in lipid biosynthesis in various organisms [39] and therefore their exact role in HSV-2 and HIV-1 replications need to be investigated further. The nuclear pore complex proteins (NPCs) promote the ferrying of nucleic acids and have been implicated in the replication of viruses including HIV-1 [40]. Of the many NPCs, it has been speculated that nucleoporin 153 (NUP153) is essential for HIV-1 replication in non-dividing cells [41]. The mRNA of this protein was upregulated by 2-fold in A3R5.7 cells infected with HSV-2 and HIV-1, along with mRNAs of NUP50, NUP54, NUP43, and NUP210. In CEM CCR5 + cells, the expression of NUP42, NUP63, and NUP210 was upregulated in HSV-2 and HIV-1 co-infected cells. These NPC proteins have been implicated in controlling the expression of various genes in the cell cycle [42]. Apart from gene expression, it has also been shown that the NPC proteins play an important role in the restriction of viral replication within the host [43]. This is a very important aspect as some of these NUPs involved can also control the fate of the cell through impact on the expression of genes controlling cell proliferation/growth arrest and regulating WNT signaling [44].

In our study, we also observed an increase in mRNA expression of several Vacuolar Protein Sorting (VPS) and Solute Carrier Proteins (SLC) in both co-infected cell lines. The VPS pathway is the central pathway to eukaryotic cells and is used to cycle various ubiquitinated receptors from the host cell surface to the lysosomal compartments via endosomes [45,46]. These proteins have been implicated in the transfer of viral proteins by targeting them to the plasma membrane and are essential for the budding of many enveloped viruses [47]. Similarly, the SLCs are some of the carrier proteins that are important in the passive transport of small molecules [48]. Besides the transport of small molecules, some SLCs also play important roles in the innate immune response. SLC15A3 is important for innate immune responses against HSV-1 and inhibition of this protein leads to enhancement of HSV-1 replication [49], which may or may not hold true for HSV-2. Studies have also shown that allele 3 of the SLC11A1 increases susceptibility to HIV-1 [50]. Reports also indicate that Hepatitis C Virus (HCV) modulates the SLC3A2 carrier for viral propagation and, while doing so, activates the Mammalian Target of Rapamycin complex 1 (mTORC1) pathway and shows increased pathogenesis [51]. Hence, understanding the influence of HSV-2 co-infection on SLCs and its effect on HIV-1 is important as many of these proteins are also known to interact with anti-retrovirals (ARVs) and help in their transport.

This study was one of a kind to assess the effect of HSV-2 on HIV-1 replication by assessing its ability to manipulate gene expression of T-cells in vitro. Studies carried out previously used a dual cell co-culture model utilizing monocyte-induced dendritic cells and T-cells from rhesus monkeys [11]. Similarly, ex-vivo studies carried out by Rollenhagen and colleagues (2014) used ex-vivo tissue models to assess the effect of HSV-2 on HIV-1 replication [52], while LeGoff et al. (2007) used HSV-2 and HIV-1 permissive CEM and P4P cell lines to study HSV-2 and HIV-1 co-infection for the generation of HIV-1/HSV-2 pseudotyped particles [53]. Only two previous studies were carried out in T-cell lines to investigate the co-existence of HSV-2 and HIV-2 in one cell [12,13]. In many of the studies reported above, researchers have used different strains of HSV-2 and, therefore, to assess if all HSV-2 strains affect replication of all HIV-1 subtypes, experiments with one subtype of HIV-1 and more strains of HSV-2 need to be carried out which may be one of the drawbacks of the study. Additionally, we have investigated the changes in gene expression at mRNA levels. In order to further confirm the data on protein interactions, studies will have to be carried out to assess the true effect of the changes observed in this study.

## 5. Conclusions

This in vitro HSV-2 and HIV-2 co-infection study carried out in immortalized T-cell lines showed that the peripheral T-cell line CEM CCR5+ and mucosal T-cell line A3R5.7 cell line is ideal for assessing the interaction of these co-infecting viral pathogens in vitro. The RNA sequencing data indicate that HSV-2 has the ability to influence early (nuclear transport and transcription) as well as late events (viral assembly and egress) in T-cell lines. Furthermore, the increase in HIV-1 viral copy number in co-infection events may be a result of increased survival of the T-cell lines due to inhibition of the WNT-signaling, apoptosis and autophagy pathway. The impact of the changes observed in this in vitro study on the outcome HIV-1 disease progression should be investigated further in a clinical study. With the ever-present threat of drug resistance in HIV-1, it would be worthwhile to assess the efficacy of known drug candidates against some of the pathways identified in this study for designing new chemical entities against HIV-1.

## Figures and Tables

**Table 1:** Number of paired-end sequences generated in mRNA sequencing.

No	Date Set	No. of Sequences in Pairs
1	A_HI	85,982,460
2	A_H	65,015,764
3	A_I	40,274,556
4	A_UI	42,505,414
5	A_CC	52,913,634
6	C_HI	50,916,060
7	C_H	63,085,772
8	C_I	68,760,056
9	C_UI	70,385,088
10	C_CC	46,314,188
	Total sequences in data sets	586,152,634 sequences
	Total nucleotides in data sets	47,019,365,884 nucleotides

Labeling infection condition: first letter (A or C) represents the cell line I = HIV-1 infection, HI = HSV-2 and HIV-1 co-infection, UI = UV-HSV-2 and HIV-1 co-infection. FDR = False Discovery rate, FC = Fold change.

**Table 2:** Changes in expression of mRNA in T-cell lines.

No	Groups	Total Gene Targets Mapped with *p* Value < 0.05 and FDR ≤ 0.05	Genes Expressed Only in the First Infection Condition	Genes Expressed Only in Second Infection Condition	No of Genes Upregulated ≥ 1.5 log_2_ (FC)	No of Genes Downregulated ≤ −1.5 log _2_(FC)
1	A-HI vs. A-I	15,460	6792	120	8152	10
2	A-UI vs. A-I	16,574	6317	321	9933	0
3	C-HI vs. C-I	12,278	497	516	1014	652
4	C-UI vs. C-I	12,264	955	991	1961	357

Labeling infection condition: first letter (A or C) represents the cell line I = HIV-1 infection, HI = HSV-2 and HIV-1 co-infection, UI = UV-HSV-2 and HIV-1 co-infection. FDR = False Discovery rate, FC = Fold change.

**Table 3:** Fold difference in the expression of genes by qPCR.

Gene ID	A-HI vs. A-I	A-UI vs. A-I	C-HI vs. C-I	C-UI vs. C-I
XPO1	1.245	0.172	−2.404	−2.294
XPO5	−1.577	−1.416	0.128	1.184
XPO7	1.047	0.203	0.159	−0.011
NUP62	1.059	0.202	0.515	0.106
NUP50	1.512	0.297	0.190	−0.173
NUP210	2.107	1.740	0.595	1.194
TSG101	−4.344	−1.955	−2.288	−3.696
VPS13A	0.485	0.163	−1.153	−1.260
VPS13C	0.995	−0.061	1.233	1.941
VPS25	0.635	0.060	1.751	1.074
WNT3	1.129	−0.172	−0.465	−1.159
TSC1	0.443	−0.201	1.694	1.475
TSC2	0.846	−0.290	−0.067	−0.243
TSC22D1	0.921	−0.572	−1.470	−2.020

Labeling infection condition: first letter (A or C) represents the cell line, H = HSV-2 infection, I = HIV-1 infection, HI = HSV-2 and HIV-1 co-infection, UI = UV-HSV-2 and HIV-1 co-infection, last letter C = cell control/mock infected cells. FDR = False discovery rate, FC = Fold change.

## Data Availability

All data are included in the manuscript and the Supplementary Material.

## Supplementary Materials

The following supporting information can be downloaded at: https://www.mdpi.com/article/10.3390/v14081715/s1, Figure S1: HSV-2 and HIV-1 viral copy number released in culture supernatants of infected T-cell line Indirect Immunofluorescence Assays of co-infected cells. Figure S2: Indirect Immunofluorescence Assays of co-infected cells. Figure S3: Log2 (RPKM) heatmap of mRNA of Cluster of Differentiation (CD)markers in CEM CCR5+ and A3R5.7 T-cell lines. Table S1: List of primers used for qPCR analysis, excel sheet S1: A_HI vs. A_I, excel sheet S2: A_UI vs. A_I, excel sheet S3: C_HI vs. C_I, excel sheet S4: C_UI vs. C_I.
