# Supplementary material for: Altered HIV-1 Viral Copy Number and Gene Expression Profiles of Peripheral (CEM CCR5+) and Mucosal (A3R5.7) T Cell Lines Co-Infected with HSV-2 In Vitro
Source: Viruses. 2022 Aug 4;14(8):1715. doi: 10.3390/v14081715 (PMC9413683; doi:10.3390/v14081715)
Supplement: Supplementary file 1 [file viruses-14-01715-s001.zip › Supplementary figures_ revised.pdf]

Supplementary information

Article

Altered HIV-1 Viral load and Gene Expression Profiles of peripheral (CEM CCR5+) and mucosal (A3R5.7) T cell lines co-infected with HSV-2 *in vitro*

Dipen Desai [1], Rajkumar Londhe [1], Madhuri Chandane[1] and Smita Kulkarni [1,*]

[1] **Division of Virology, ICMR-National AIDS Research Institute, Pune 411026, Maharashtra,**

India

*** Correspondence: skulkarni@nariindia.org**

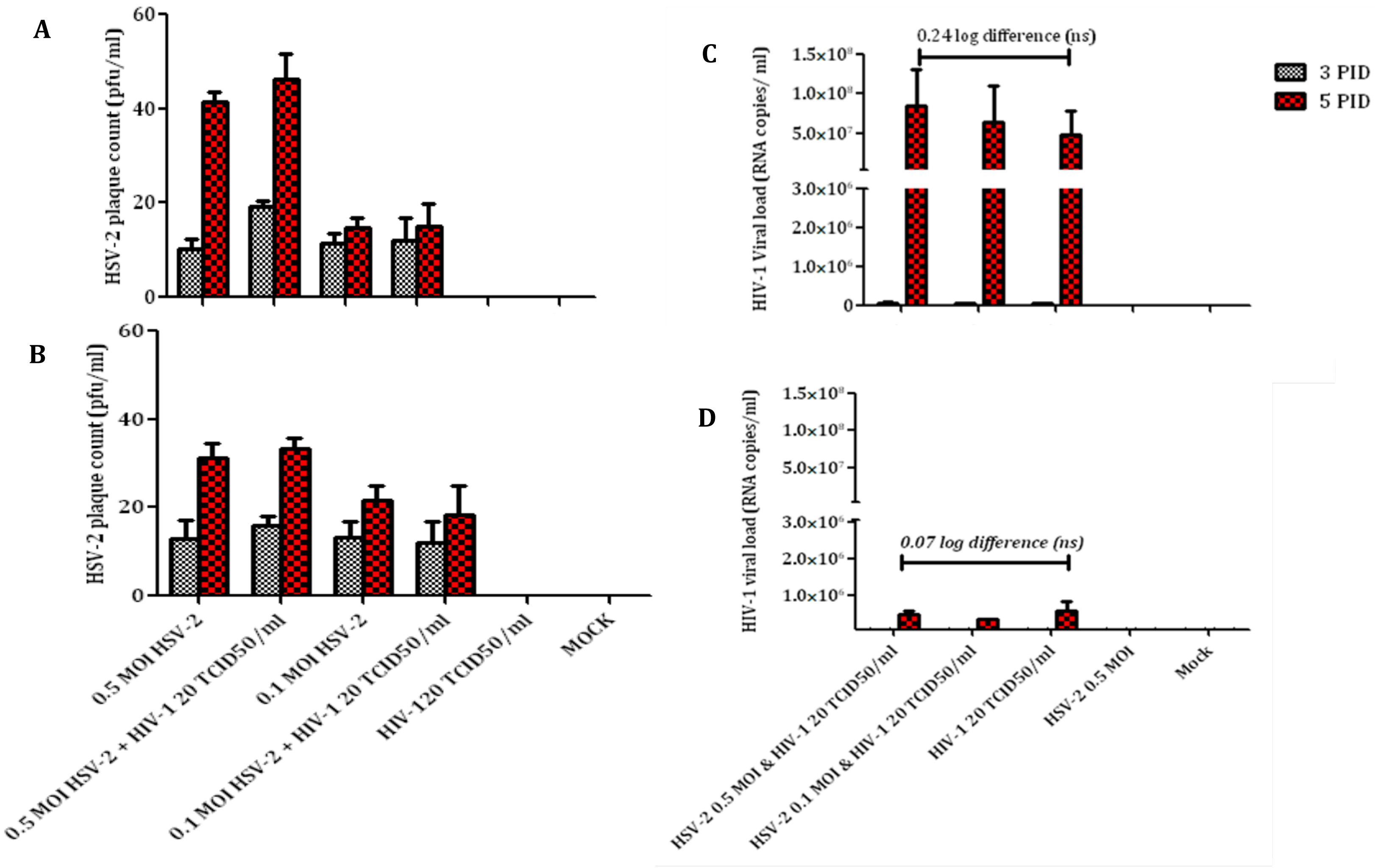

Figure S1: HSV-2 and HIV-1 viral copies released in culture supernatants of infected T-cell lines.
HSV-2 plaque counts expressed as pfu/ml in PM-1(A) and MOLT4 CCR5+ (B) T-cell lines in co-infection assays. Plaques were counted using Vero cell line. HIV-1 viral cop[ies in culture supernatants PM-1(C) and MOLT4 CCR5+ (D) as estimated by Abbott automated real-time PCR. Data represents Mean ± SD of three independent assays.

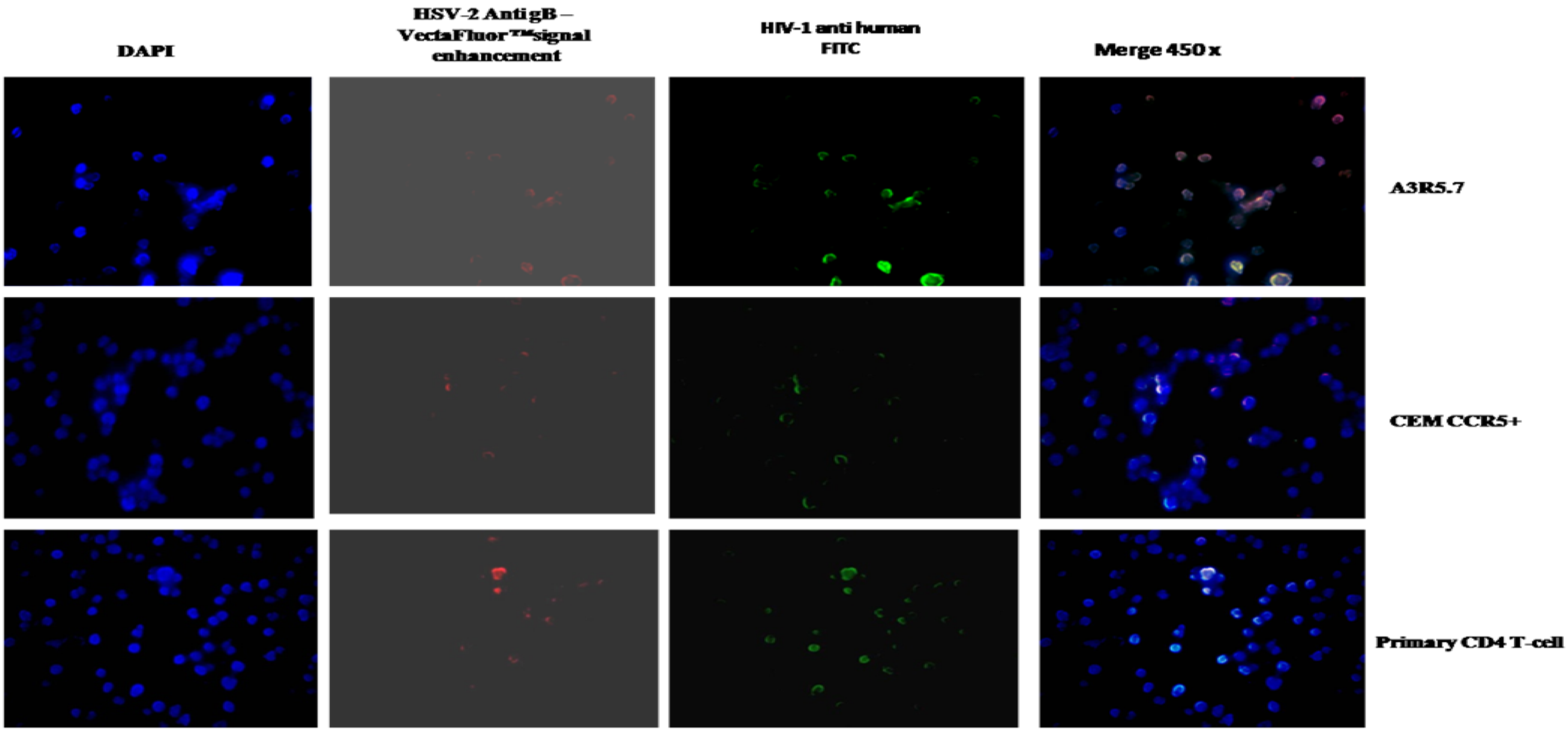

Figure S2. In-direct immunofluorescence assay of HSV-2 and HIV-1 co-infected T-cells

Fixed T-cells (A3R5.7, CEM CCR5+ and primary CD4+ T-cells) were treated anti- HSV-2 gB (mouse monoclonal) and HIV-1 positive human serum (polyclonal) and counterstained with goat anti-mouse VectafluorTM enhancement kit (Trit-C, Red) and anti Human-FITC (Green) antibodies. Nucleus was stained using DAPI (blue). Cells visualized under Olympus X51 fluorescent microscope with a total magnification of 450 X and images combined using the CellF software.

A

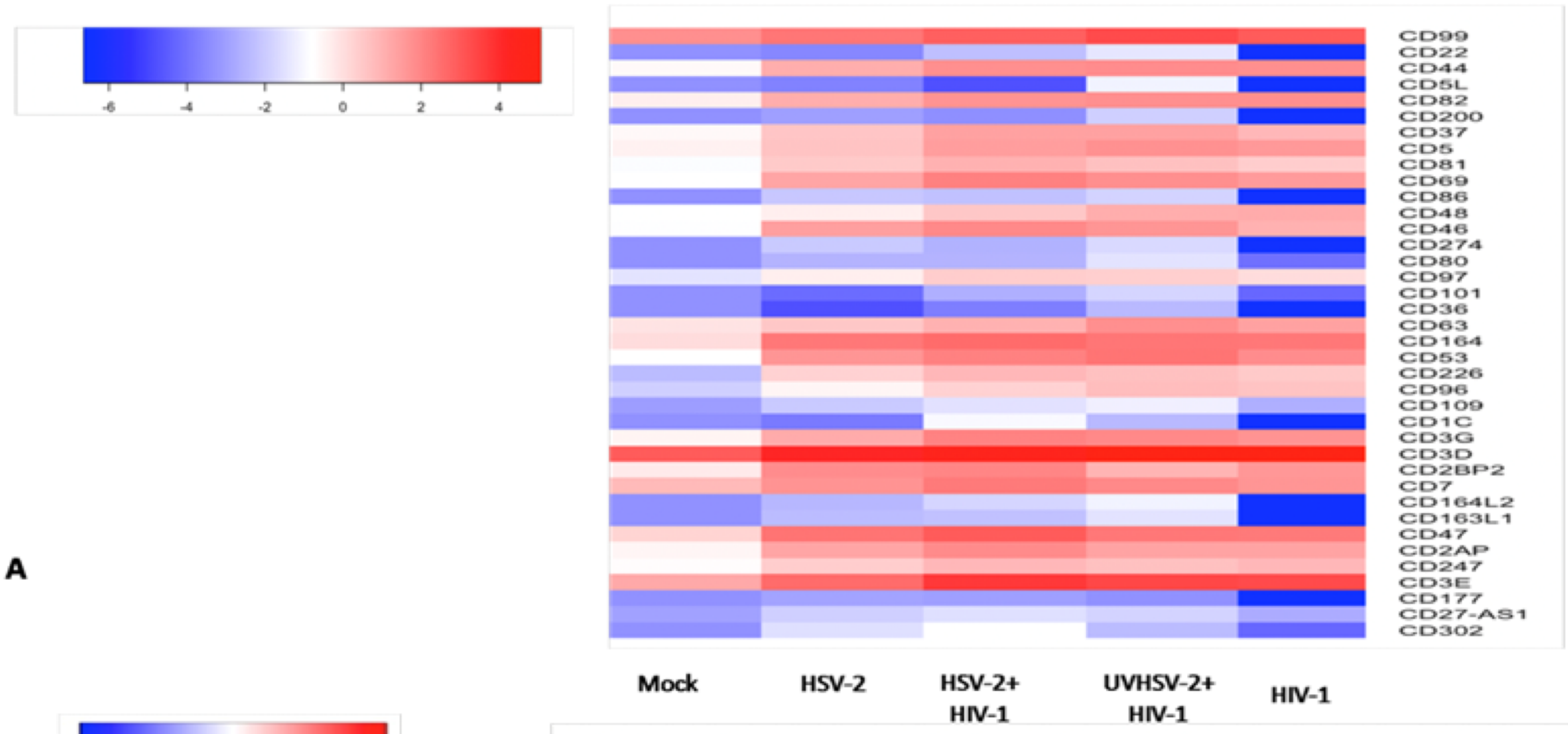

B

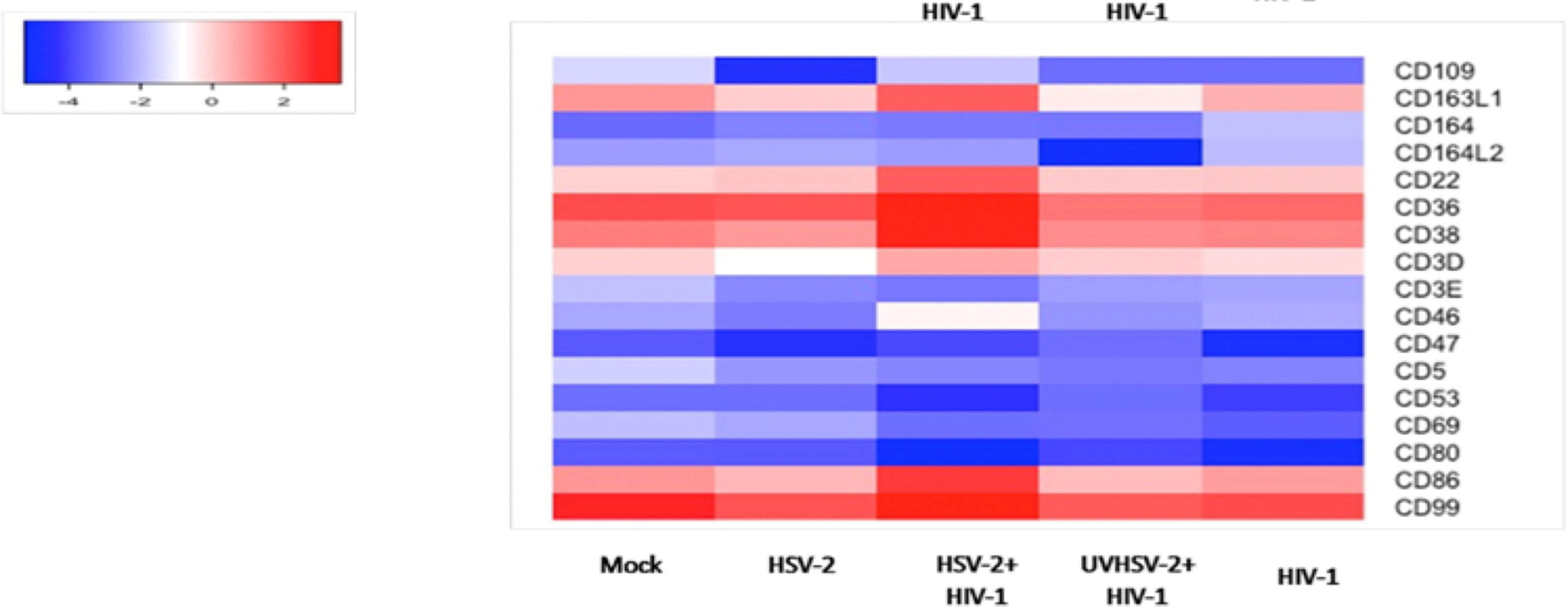

Figure S3. Log2 (RPKM) values of T-cell lines (A3R5.7 and CEM CCR5+) infected under various conditions were used to draw a heatmap using the ggplots2 program on R-studio. A. Heatmap generated for A3R5.7 cells B. Heatmap generated for CEM CCR5+ T-cell line.

Table S1. List of primers used for qPCR analysis.

No	GENE ID	forward primer	reverse primer
1	XPO1	GGCATTTCGTTCAGGTTCAG	ACCAATCATGTACCCCACAG
2	XPO5	TGCTGAAAACTGTAAACTCCTG	TCTACCAAACCTCCTCCATC
3	XPO7	TCTTATCTCCACCCTCTTTCC	TCACACAGACCAAGAACCC
4	NUP62	TCCAACCAGCCACAAGTACC	TCCCCAAAGAAAATCCAGTTCC
5	NUP210	ACATCCCTCCTTCTTACATCTC	ACATTCTTGTAGACAGCCTCC
6	NUP50	AACCTACAGCAAATCAGAAGAC	TTGGAACACAGACGATAAGAAC
7	WNT3	ACCTCAAATGCAAGTGCC	TGCTTCTCTACTACCATCTCC
8	TSG101	CAGGGAACTAATGAACCTCAC	CGAAATAGGACGAGAGAAGAC
9	VPS13A	CTTTTCTGCCCCCAAAATAAAC	GCATAAGCCCACCATTCTC
10	VPS25	AGTATCGCTTCCCACCCTTC	GTTCCCTTTCTTCCTCAGTTCC
11	VPS13C	TACCTGCTTGTTGTCCCTG	GCCTTTTTCTGCTGCTTTTTG
12	TSC1	GGTCAAGCCAATGATGGAG	GTGGAGTAAGGGGTAGAAGTAG
13	TSC2	AGGGGCAAGAGAGTAGAGAG	AAAGAAGGGGGAATGGTAGAG
14	TSC22D1	AGCAGGTAAGAGAAGAGACAG	AAAGCCAAAGGGGAATGTAAG
15	GAPDH	CATGAGAAGTATGACAACAGCC	TGAGTCCTTCCACGATACC
